# The prognostic value of the MASS in a multi-center cohort of patients with newly diagnosed multiple myeloma

**DOI:** 10.1038/s41408-022-00731-4

**Published:** 2022-09-14

**Authors:** Peiyu Yang, Fan Zhou, Yujun Dong, Guangxun Gao, Hua Xue, Xinyue Liang, Shanshan Yu, Weiling Xu, Yanping Ma, Xiaoqi Qin, Mengyao Li, Yun Dai, Fengyan Jin

**Affiliations:** 1 Department of Hematology, the First Hospital of Jilin University, Changchun, Jilin China; 2 Laboratory of Cancer Precision Medicine, the First Hospital of Jilin University, Changchun, Jilin China; 3 Department of Hematology and Oncology, Shanghai Jing’an District Zhabei Central Hospital, Shanghai, China; 4 Department of Hematology, Peking University First Hospital, Beijing, China; 5 Department of Hematology, Xijing Hospital, Air Force Medical University, Xi’an, Shanxi China; 6 Department of Hematology, the Affiliated Hospital of Hebei University, Baoding, Hebei China; 7 Department of Radiology, the First Hospital of Jilin University, Changchun, Jilin China; 8 Department Hematology, the Second Hospital of Shanxi Medical University, Taiyuan, Shanxi China

**Keywords:** Myeloma, Disease-free survival

Dear Editor,

Multiple myeloma (MM) emerges as a heterogeneous disease with a considerable diversity in tumor biology, clinical characteristics, therapeutic responses, and outcomes in the era of novel agents and therapies (e.g., proteasome inhibitors/PI, immunomodulatory drugs/IMiD, CD38 monoclonal antibodies, etc.) [1]. To guide making treatment decision, multiple risk stratification systems have been developed to discriminate different risk levels of MM patients at diagnosis, including the ISS or its successor R-ISS currently used in clinical practice [2]. The R-ISS was created via updating the ISS by including elevated lactate dehydrogenase (LDH) and high-risk cytogenetic abnormalities (HRCA) such as del(17p), t(4;14), and t(14;16) [3, 4]. However, with the paradigm shift in the treatment of MM, the impact or weight of these and other baseline risk factors in estimating the outcomes of MM patients may have changed [5]. For example, the prognostic impact of some additional HRCAs that have not been included in the R-ISS [6, 7], such as +1q including 1q21 gain (3 copies) or amplification (≥ 4 copies) [8, 9], as well as their concurrence in various combinations [10] (so called double- and triple-hit [11]) have been emerging. Moreover, one of the limitations for the R-ISS is that more than a half of patients with newly diagnosed MM (NDMM) have been classified as R-ISS II with intermediate risk, whose outcomes may however vary to a large extent. To address these concerns, two staging systems, including the Mayo Additive Staging System (MASS) and Second Revision of the International Staging System (R2-ISS), have been reported very recently [12, 13].

These two new algorithms have been built on virtually same risk factors that are associated with overall survival (OS; the MASS) or both progression-free survival (PFS) and OS of NDMM patients (the R2-ISS), including ISS III (ISS II was also scored in the R2-ISS), elevated LDH, del(17p), +1q, and HR IGH translocation [12, 13]. The MASS includes any HR IGH translocation, while the R2-ISS only included t(4;14). Although t(14;16) is demonstrated as an independent adverse factor for OS, it is not included in the R2-ISS because its effect on PFS was not statistically significant [13]. +1q is scored as 1 (same as other HRCAs) in the MASS while 0.5 (lower than other HRCAs) in the R2-ISS. The MASS stratifies NDMM patients more evenly into MASS I (36%), II (33%), and III (31%) than the R-ISS (17%, 66%, and 17% for R-ISS I, II, and III, respectively) [12]. The performance of the MASS and R2-ISS is comparable in re-stratification of R-ISS I patients to I (70–80%) and II stages (20–30%). The MASS re-stratify R-ISS II patients to MASS I (32%), II (47%), and III (21%), while the R2-ISS discriminates R-ISS II patients with R2-ISS II (38%), III (59%), and IV (3%). Unlike the R2-ISS that re-stratifies R-ISS III patients to R2-ISS III (41%) and IV (59%), all R-ISS III patients remain as MASS III. Nonetheless, the performance of these new staging systems requires further validation, especially considering the dissimilarity of MM patients among different populations.

Here, we sought to test the prognostic value of the MASS by analyzing our clinical retrospective data of patients diagnosed with MM between 27 November 2009 and 20 November 2019 at seven centers nationwide in China. All patients must have baseline information available for the MASS scoring, particularly cytogenetics by FISH that must include the probes for del(17p), 1q+, and HR IGH translocations [t(4;14) and t(14;16)]; they must receive novel agents (PI, IMiD, or both) for first-line treatment. According to the MASS that scores each risk factor as 1, patients were divided into three groups, including MASS I (score 0), II (score 1), and III (score ≥ 2) [12]. PFS was defined as the time from diagnosis until disease progression, relapse, or death due to any cause. Patients who did not progress or relapse were censored on the last date when they were seen alive and event free. OS was defined as the time from diagnosis until death due to any cause or last followup. This study was approved by the Institutional Review Board (IRB) of the First Hospital of Jilin University (Approval # 2016-087). All patients had given written informed consent to the use of clinical data according to the Declaration of Helsinki.

In this cohort (*n* = 1005), there were clearly more patients with advanced diseases (e.g., ISS and R-ISS III), large tumor burden (e.g., elevated LDH and β2-MG), and organ involvement (e.g., CRAB), compared to the Mayo cohort (Table 1). Notably, the frequency of +1q was higher (51.8%) in this cohort as we observed earlier in Chinese NDMM patients [14], while del(17p) and HR IGH translocation were comparable between these two cohorts. First-line treatment included PI (51% vs. 31%), IMiD (18% vs. 31%), or both (32% vs. 34%), but much less patients received transplant in this cohort (12% vs. 55%), mostly due to unaffordability. According to the IMWG consensus criteria [15], 114 (12.4%), 207 (22.6%), 244 (26.6%), 235 (25.6%), 58 (6.3%), and 59 (6.4%) patients had sCR, CR, VGPR, PR, MR, and SD, respectively. With median follow-up of 35.5 months (95% CI, 32.8–38.2), median PFS and OS were 25.2 (95% CI, 23.1–27.3) and 53.0 (95% CI, 48.1–57.9) months.

**Table 1:** Comparison of baseline characteristics between two cohorts.

Characteristics	Our cohort, *n* (%)	Mayo cohort [12]
Age (yrs), median (range)	61 (27–89)	64 (57–71)
Sex, male	590 (58.7)	62%
M protein		
IgG	452 (45.0)	62%
IgA	249 (24.8)	25%
IgD	61 (6.1)	–
LC	215 (21.4)	11%
Non/oligosecretory	27 (2.7)	–
Biclonal	1 (0.0)	–
ISS stage		
I	169 (16.8)	–
II	306 (30.4)	–
III	530 (52.7)	33%
R-ISS stage		
I	118 (11.7)	11%
II	624 (62.1)	66%
III	263 (26.2)	23%
LDH, elevated	265 (26.4)	17%
BMPCs, ≥ 30% (*n* = 562)	358 (63.7)	50 (30–70)^a^
β2-MG, ≥ 5.5 μg/ml (*n* = 567)	324 (57.1)	32%
Hemoglobin, ≤10 g/dL (*n* = 948)	635 (67.0)	33%
Calcium, ≥ 1 mg/dL (*n* = 1003)	143 (14.3)	11%
Creatinine, ≥2 mg/dL (*n* = 1004)	261 (26.0)	16%
Bone disease (*n* = 963)	887 (92.1)	–
Extramedullary lesion (*n* = 960)	193 (20.1)	–
Albumin, <3.5 g/dL (*n* = 899)	523 (58.2)	48%
Platelet, <100 × 10^9^/L (*n* = 1002)	149 (14.9)	210 (162–262)^a^
+1q	521 (51.8)	31%
del(17p)	113 (11.2)	13%
del(13q) (*n* = 978)	412 (42.1)	37%^b^
del(1p) (*n* = 413)	35 (8.5)	–
IgH translocation		
t(11;14)	130 (12.9)	21%
t(4;14)	138 (13.7)	10%
t(14;16)	22 (2.2)	4%
First-line treatment		
PI	511 (50.8)	31%
IMiD	177 (17.6)	31%
PI + IMiD	317 (31.5)	34%
Transplant	122 (12.1)	55%

*LC* light chain, *BMPCs* bone marrow plasma cells, *β2-MG* β2-macroglobulin, *LDH* lactate dehydrogenase, *PI* proteasome inhibitor, *IMiD* immunomodulatory drug.

^a^Median (range).

^b^Monosomy 13.

According to the MASS, all patients could be stratified to MASS I with no risk factor (170,16.9%), II with one risk factor (330, 32.8%), and III with ≥ 2 risk factors (505, 50.3%). Compared to the Mayo cohort [12], there were relatively less patients with early stage disease (MASS I) but more patients with late stage disease (MASS III), consistent with the fact that majority of patients had advanced disease in this cohort. For MASS I, II, and III, median PFS was 45.6, 27.4, and 20.3 months; and median OS 88.3, 62.9, and 40.6 months, respectively. For each stage, the outcomes of patients were worse than those in the Mayo cohort, while the differences in both PFS (Fig. 1a) and OS (Fig. 1b) were significant for MASS I vs. II (PFS: HR, 1.732; 95% CI, 1.313–2.284; *P* = 0.0001; OS: HR, 1.647; 95% CI, 1.120-2.422; *P* = 0.0111) or II vs. III (PFS: HR, 1.505; 95% CI, 1.256–1.802; *P* < 0.0001; OS: HR, 1.934; 95% CI, 1.530–2.443; *P* < 0.0001). Together, these observations support the value of the MASS in risk stratification of NDMM patients at diagnosis and prediction of both PFS and OS.

**Fig. 1:**
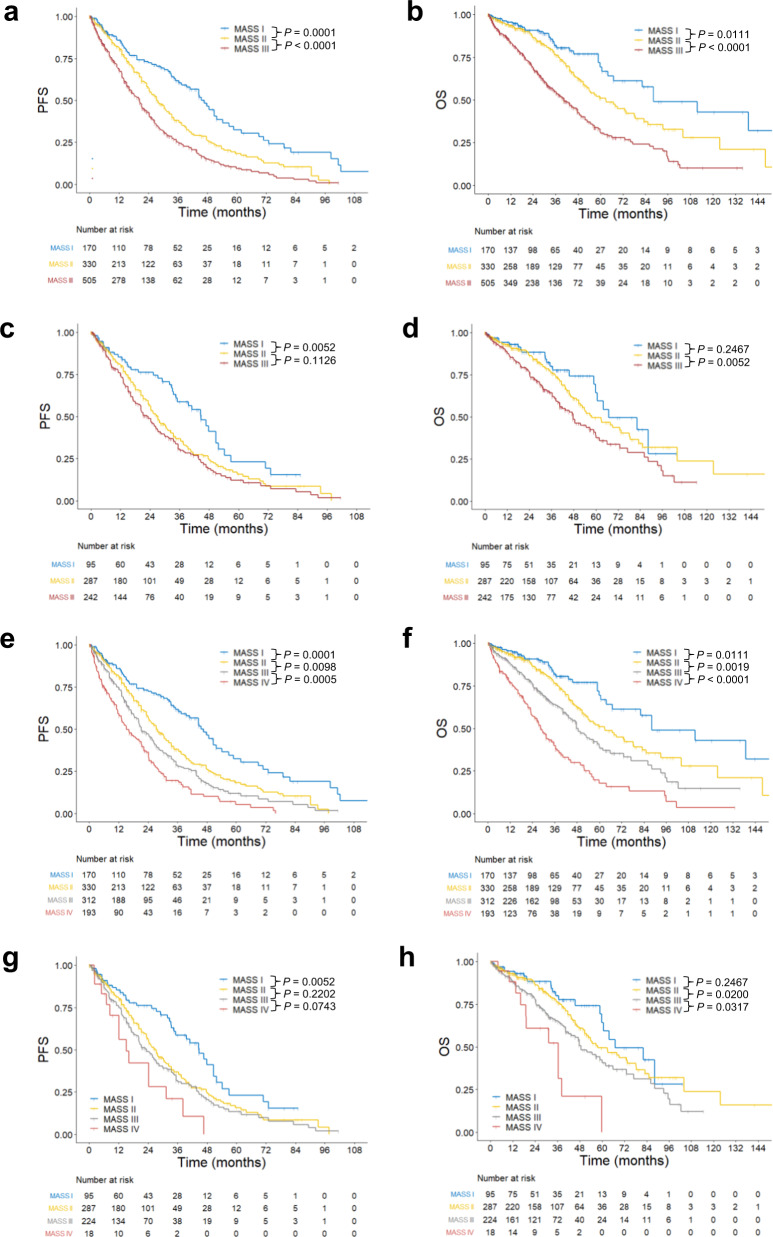
Survival of patients with NDMM based on the MASS. PFS **a** and OS **b** in patients (*n* = 1005) with stage I (total score = 0 point), II (total score = 1 point), and III (total score ≥ 2 points) determined by the 3-tier MASS, in which each high-risk factor (i.e., high-risk IGH translocation, 1q gain/amplification, chromosome 17 abnormality, ISS stage III, or elevated LDH) scored one point. PFS **c** and OS **d** in R-ISS II patients (*n* = 624) with stage I, II, and III determined by the 3-tier MASS. PFS **e** and OS **f** in patients (*n* = 1005) with stage I (total score = 0 point), II (total score = 1 point), III (total score = 2 points), and IV (total score ≥ 3 points) determined by the 4-tier MASS, in which each high-risk factor described above scored one point. PFS **g** and OS **h** in R-ISS II patients (*n* = 624) with stage I, II, III, and IV determined by the 4-tier MASS.

The MASS seems to stratify patients with intermediate risk (e.g., R-ISS II, accounting for about 60% of patients with heterogeneous outcomes) better than the R-ISS [12]. In 624 R-ISS II patients, the MASS further stratified them to MASS I (95,15.2%), II (287, 46.0%), and III (242, 38.8%), with median PFS of 44.7, 27.3, and 22.8 months (Fig. 1c), and median OS of 67.0, 57.8, and 47.7 months (Fig. 1d), respectively. The differences in PFS were significant for MASS I vs. II (HR, 1.644; 95% CI, 1.160–2.331; *P* = 0.0052), but not II vs. III (HR, 1.198; 95% CI, 0.958–1.497; *P* = 0.1126). By contrast, the differences in OS were significant for MASS II vs. III (HR, 1.505; 95% CI, 1.130–2.005; *P* = 0.0052), but not I vs. II (HR, 1.319; 95% CI, 0.826–2.105; *P* = 0.2467). Moreover, the MASS re-stratified R-ISS I patients (*n* = 118) to MASS I (75, 63.6%) and II (43, 36.4%), while all R-ISS III patients (*n* = 263) were MASS III. Therefore, these observations verify the notion that R-ISS II patients had heterogeneous outcomes, which could, at least in part, be discriminated by the MASS.

The MASS can also been used as a 4-tier staging system [12]. In this case, 1005 patients were stratified into MASS I (170,16.9%), II (330, 32.8%), III (312, 31%), and IV (193, 19.2), with median PFS of 45.6, 27.4, 21.3, and 15.2 months, and median OS of 88.3, 62.9, 48.7, and 28.7 months, respectively. This 4-tier version of the MASS could further stratify patients with MASS III (3-tier) to III and IV on both PFS (Fig. 1e**;** HR, 1.485; 95% CI, 1.189–1.854; *P* = 0.0005) and OS (Fig. 1f**;** HR,1.890; 95% CI, 1.458–2.450; *P* < 0.0001**)**. Moreover, it also further stratified R-ISS II patients (*n* = 624) with MASS III (3-tier) to III (224, 35.9%) and IV (18, 2.9%), with median PFS of 23.3 vs. 15.1 months (Fig. 1g; HR, 1.656; 95% CI, 0.952–2.880; *P* = 0.0743) and median OS of 48.7 vs. 36.5 months (Fig. 1h; HR, 2.004; 95% CI, 1.063–3.779; *P* = 0.0317). These findings suggest that the 4-tier MASS might perform better than the 3-tier one in risk stratification, at least in this cohort of NDMM patients with more advanced disease and worse outcomes.

In conclusion, this study provides additional evidence supporting the prognostic value of the MASS in risk stratification of NDMM patients at diagnosis, particularly those with R-ISS II defined by the R-ISS, in an entirely independent cohort involving Chinese patient population. Thus, this new simple additive staging system warrants further attention in future investigation and daily practice.

## Data Availability

Original data are available to other investigators upon request.
